# *Streptomyces roseolus*, A Promising Biocontrol Agent Against *Aspergillus flavus*, the Main Aflatoxin B_1_ Producer

**DOI:** 10.3390/toxins10110442

**Published:** 2018-10-30

**Authors:** Isaura Caceres, Selma P. Snini, Olivier Puel, Florence Mathieu

**Affiliations:** 1 Laboratoire de Génie Chimique, Université de Toulouse, CNRS, INPT, UPS, 31326 Toulouse, France; isauracrl1@gmail.com (I.C.); selma.snini@ensat.fr (S.P.S.); 2 Toxalim (Research Center in Food Toxicology), Université de Toulouse, INRA, ENVT, INP-Purpan, 31300 Toulouse, France; olivier.puel@inra.fr

**Keywords:** *Aspergillus flavus*, *Streptomyces roseolus*, biocontrol, aflatoxin B_1_, gene expression, fungal morphology

## Abstract

Crop contamination by aflatoxin B_1_ is a current problem in tropical and subtropical regions. In the future, this contamination risk may be expanded to European countries due to climate change. The development of alternative strategies to prevent mycotoxin contamination that further contribute to the substitution of phytopharmaceutical products are thus needed. For this, a promising method resides in the use of biocontrol agents. Several actinobacteria strains have demonstrated to effectively reduce the aflatoxin B_1_ concentration. Nevertheless, the molecular mechanism of action by which these biological agents reduce the mycotoxin concentration has not been determined. The aim of the present study was to test the potential use of *Streptomyces roseolus* as a biocontrol agent against aflatoxin B_1_ contamination. Co-cultures with *Aspergillus flavus* were conducted, and the molecular fungal response was investigated through analyzing the q-PCR expression of 65 genes encoding relevant fungal functions. Moreover, kojic and cyclopiazonic acid concentrations, as well as morphological fungal changes were also analyzed. The results demonstrated that reduced concentrations of aflatoxin B_1_ and kojic acid were respectively correlated with the down-regulation of the aflatoxin B_1_ gene cluster and *kojR* gene expression. Moreover, a fungal hypersporulated phenotype and a general over-expression of genes involved in fungal development were observed in the co-culture condition.

## 1. Introduction

*Aspergillus flavus* is an opportunist soil pathogen fungus that is implicated in contamination issues in the agriculture field and causes important economic losses [1]. This species is commonly found in crops such as maize, soybean, as well as oilseed, peanuts, dried fruits, and spices. *A. flavus* produces a large number of secondary metabolites including several mycotoxins which impact the food sanitary quality. In this context, aflatoxin B_1_ (AFB_1_) is recognized as the most potent naturally occurring carcinogenic agent [2]. Indeed, AFB_1_ has been demonstrated to induce liver cancer and immunotoxic effects in humans and animals as well as growth impairment in children [3,4]. Due to the physiological properties of *A. flavus*, AFB_1_ contamination is mostly found in tropical and subtropical regions. However, due to climate change, the geographical distribution of AFB_1_ contamination may increase in future years, leading to the occurrence of this mycotoxin in areas considered safe until now [5,6]. Contamination by aflatoxins frequently occurs with cyclopiazonic acid (CPA), another mycotoxin commonly produced by *A. flavus* strains that was demonstrated to be a cytotoxic agent in humans [7,8]. *Aspergillus* section *Flavi* species are also able to synthesize non-toxinogenic secondary metabolites, such as kojic acid (KA), a metabolite of interest for diverse industries. For example, KA is employed as an inhibitor of pigment formation in plant and animal tissues in order to preserve or change a substance’s color [9]. Currently, the use of phytopharmaceutical products is the most frequent strategy to limit mycotoxin contamination. However, the massive use of such products has led to the accumulation of toxic chemical residues in agricultural products as well as in water and soil [10]. This observation led researchers to develop alternative strategies such as the use of biocontrol agents. In fact, certain microorganisms have demonstrated the ability to reduce mycotoxin concentrations while maintaining an ecological niche balance [11,12]. Concerning AFB_1_ biocontrol, one of the best-known strategies is the use of non-aflatoxigenic *A. flavus* strains, like Afla-guard^®^ (Circle One Global, Inc., Shellman, GA, USA) or Aflasafe^®^ (IITA, Ibandan, Nigeria). However, it has been demonstrated that recombination phenomena between non-aflatoxigenic and aflatoxigenic *A. flavus* strains can occur, decreasing the effectiveness of this method [13,14]. In parallel, bacterial strains belonging to the *Bacillus*, *Pseudomonas*, *Agrobacterium and Streptomyces* genera have also been demonstrated to limit mycotoxin concentrations [15,16,17,18]. Within this group, *Streptomyces* spp are ubiquitous soil bacteria that are able to produce endospores and bioactive compounds with a broad spectrum, including anti-aflatoxinogenic properties. Recent studies have demonstrated that several actinobacteria strains greatly reduce the AFB_1_ concentration without affecting fungal growth [19,20]. Nonetheless, the specific molecular mechanism of action by which these microorganisms act remains to be determined. Therefore, the present work aimed to test the potential use of *S. roseolus* as a biocontrol agent against AFB_1_ contamination using the co-culture method with *A. flavus*. For this, analyses of fungal secondary metabolite concentrations (AFB_1_, CPA, and KA), fungal gene expressions, and morphological changes were performed. A total of 65 genes were analyzed: 29 genes involved in AFB_1_, CPA, and KA biosynthesis and 36 genes coding for diverse regulatory factors linked to fungal processes such as cellular signaling, development regulation, global transcription factors as well as genes involved in environmental and oxidative stress responses. The results demonstrated that under co-culture conditions, the AFB_1_ concentration was reduced to undetectable levels and almost all genes belonging to the AFB_1_ cluster were down-regulated. The KA concentration was reduced by 37% under the co-culture condition, and the expression of the specific transcription factor *kojR*, which is involved in KA biosynthesis, was down-regulated. The CPA concentration was not modified and the expression of the *dmaT* gene involved in its biosynthesis was up-regulated. Concerning fungal development, a hypersporulation phenotype was observed in *A. flavus*. Moreover, a general over-expression of genes involved in the regulation of fungal development was observed.

## 2. Results

### 2.1. Production of Secondary Metabolites in A. flavus

AFB_1_, KA, and CPA concentrations were quantified in both culture conditions. The co-culture condition induced a significant reduction in the concentration of AFB_1_ to undetectable levels in comparison with the control (972.8 µg/L ± 254.9 µg/L, *p*-value < 0.0001). In the co-culture condition, the HPLC analysis demonstrated a reduction in the KA concentration of 37% (1.29 mg/mL ± 0.22 mg/mL for control vs 0.81 mg/mL ± 0.29 mg/mL for co-culture conditions, *p*-value = 0.2159). Finally, the CPA concentration was slightly, but not significantly, reduced (0.79 mg/mL ± 0.08 mg/mL for the control culture vs 0.68 mg/mL ± 0.22 mg/mL for the co-culture, *p*-value = 0.6835).

### 2.2. Morphological Changes

**Figure 1:**
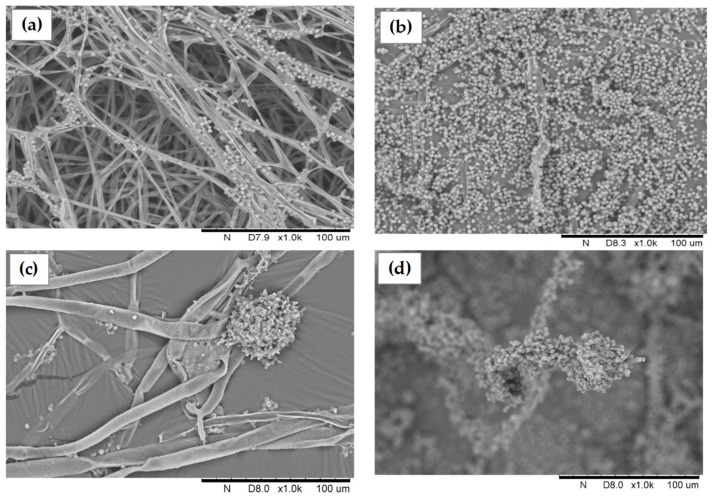
Morphological analysis of *A. flavus* by Scanning Electronic Microscopy. (**a**) Aerial mycelium in control condition; (**b**) aerial mycelium in co-culture condition; (**c**) basal mycelium in control condition and (**d**) basal mycelium in co-culture condition.

Morphological studies were performed by SEM observation and spore quantification. SEM images demonstrated that *A. flavus’* mycelium presented noticeable differences under co-culture condition. A visual augmentation in spore quantity was observed in different areas and by several prints over the aerial fungal mycelium (Figure 1a,b). Moreover, in the basal mycelium, aberrant spore development all along the hyphae was observed in the co-culture condition (Figure 1c,d). In order to confirm this phenomenon, spore quantification was performed demonstrating that the co-culture condition induces a significant 36% increase in the spore quantity in *A. flavus* (9.4 × 10^6^ ± 4.35 × 10^5^ vs. 1.2 × 10^7^ ± 3.63 × 10^5^; *p*-value = 0.004).

### 2.3. Expression Analysis of Genes Involved in Fungal Secondary Metabolite Pathways

**Figure 2:**
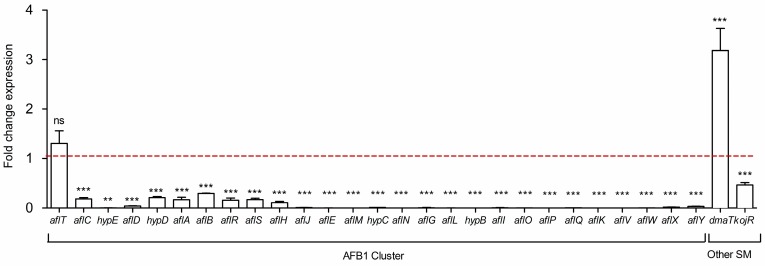
Fold change in the expression of genes belonging to the aflatoxin B_1_ (AFB_1_), cyclopiazonic acid and kojic acid biosynthetic pathways. The dotted baseline represents the control expression level; ns = no significant change; * *p*-value < 0.05; ** *p*-value < 0.01; *** *p*-value < 0.001. SM = secondary metabolite.

The AFB_1_ biosynthetic pathway is governed by 27 genes grouped into a cluster regulated by two specific transcriptional factors, *aflR* and *aflS.* In this study, the entire gene cluster was analyzed. The results demonstrated that a 4-day co-culture with *S. roseolus*, induced a significant down-regulation of all genes involved in AFB_1_ gene cluster in *A.flavus* with the only exception being *aflT* (*p*-value = 0.254) (Figure 2). The gene expression of the two specific transcriptional factors, *aflR* and *aflS*, was decreased by 6-fold. Kojic acid production is directed by a gene cluster governed by the specific transcriptional factor encoded by *kojR*. The expression of the latter was significantly reduced by 2-fold in the co-culture condition. Besides, the expression of the dimethylallyltryptophan synthase *dmaT*, involved in the CPA biosynthetic pathway, was significantly up-regulated by 3.2-fold in the co-culture condition.

### 2.4. Expression Analysis of Genes Involved in Fungal Development

**Figure 3:**
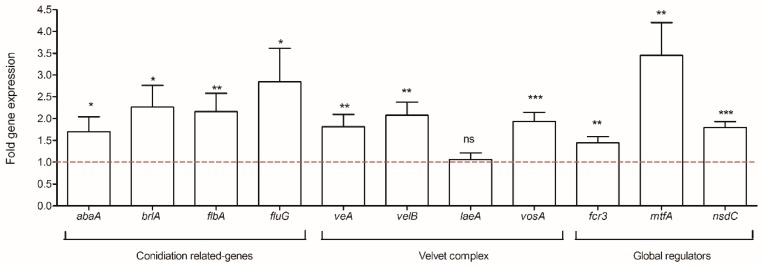
Analysis of the expression of genes linked to the conidiation process, the velvet protein complex, and global regulators. The dotted baseline represents control expression level; ns = no significant change; * *p*-value < 0.05; ** *p*-value < 0.01; *** *p*-value < 0.001.

An expression analysis of several genes involved in fungal development, the velvet family, and global regulators was performed. As shown in Figure 3, a general over-expression of the analyzed genes was observed. For instance, genes involved in conidiation and conidiophore morphology, like *abaA*, *brlA*, *flbA* and *fluG*, increased by 1.7- to 2.8-fold. Concerning the velvet family, the results showed that *veA*, *velB*, and *vosA* presented significant increases of 1.8-, 2.1-, and 1.9-fold, respectively. Nevertheless, *laeA* expression was not impacted by the co-culture condition (*p*-value = 0.710). Among the global regulators analyzed in this group, the transcription factor *mtfA* was the most affected gene with an up-regulation of 3.5-fold. Small but significant changes were also observed for *nsdC* expression, another transcription factor required for conidiophore development with an up-regulation of 1.7-fold. 

### 2.5. Expression Analysis of Genes Involved in Fungal Response to External Stimuli

**Figure 4:**
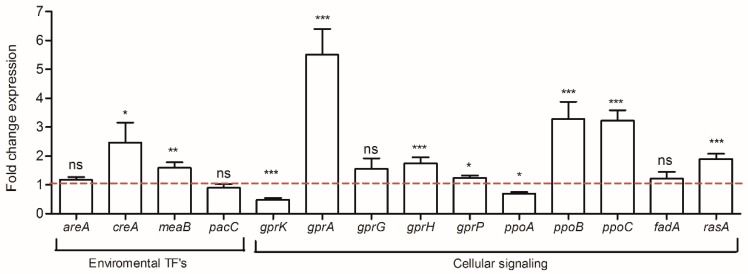
Fold change expression of genes involved in environmental responses and cellular signalization. The dotted baseline represents the control expression level; ns = no significant change; * *p*-value < 0.05; ** *p*-value < 0.01; *** *p*-value < 0.001.

In fungi, external changes trigger the rapid activation of environmental and cellular signaling transduction in order to ensure fungal adaptation. Thus, the expression of several genes involved in this process were analyzed. The results presented in Figure 4 demonstrate that the co-culture condition induced an up-regulation of the expression of *creA* and *meaB* genes by 2.5- and 1.6-fold, respectively. Among the *GPCR* genes, *gprK*, the principal regulator of the G-protein signaling presented a reduced expression by 2-fold. Despite the *gprK* down-regulation, the other *gpcr* genes were significantly up-regulated, *gprA* being the most impacted gene with an increased level by 5.5-fold. The expression of *gprG* as well as *fadA*, which encodes the subunit G-protein, did not present significant differences (*p*-values = 0.132 and 0.710). Finally, within dioxygenase oxylipins’ gene group, *ppoA* was marginally down-regulated (fold expression of 0.7) while *ppoB* and *ppoC* were up-regulated by 3.2-fold.

### 2.6. Expression Analysis of Genes Involved in Fungal Oxidative Stress Response

**Figure 5:**
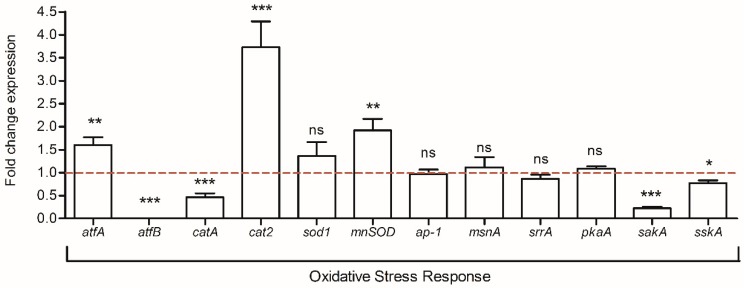
Fold change expression of genes involved in the oxidative stress response. The dotted baseline represents control expression level; ns = no significant change; * *p*-value < 0.05; ** *p*-value < 0.01; *** *p*-value < 0.001.

The response to oxidative stress is a fungal defense mechanism linked to secondary metabolite production and involves several transcription factors. Among them, the expression of 10 genes was analyzed. As shown in Figure 5, the expression levels of seven genes were significantly modified upon co-culture condition. The two most impacted genes were the bZIP transcription factor *atfB* which was dramatically down-regulated by 149.2-fold and the *cat2* gene, which codes for a bifunctional catalase peroxidase and was up-regulated by 3.7-fold. Among the other analyzed genes, *catA*, which encodes a development regulating catalase, and *sakA* and *sskA*, which are involved in the MAPK and SakA/HogA stress signaling pathways, were down-regulated by 2.2-, 4.5-, and 1.3-fold respectively. Conversely, the manganese superoxide dismutase *mnSOD* was up-regulated by 1.9-fold. Otherwise, no significant changes were observed for the rest of the analyzed genes (*p*-values = 0.210-*sod1*; 0.756-*ap1*; 0.595-*msnA*; 0.172-*srrA*, and 0.314-*pkaA*). 

## 3. Discussion

The contamination of foodstuffs by fungal development has many negative consequences such as the alteration of commodities, the loss of nutritional qualities, a strong reduction in production yields, and the accumulation of toxic compounds such as mycotoxins. For example, AFB_1_, produced by *A. flavus*, is the most potent naturally occurring carcinogen known to contaminate foodstuffs. Faced with mycotoxin contamination issues, the use of phytopharmaceutical products was favored for several years. However, consumers are increasingly paying attention to the consequences of using such chemical products in agriculture. Thus, environmental-friendly alternative methods should be developed. Within this context, in the present study, *S. roseolus* was used as biocontrol agent to reduce AFB_1_ contamination by *A. flavus* and the molecular mechanism of action was elucidated by transcriptomic analysis.

### 3.1. Effects of S. roseolus on AFB_1_ Concentration and Gene Expression of AFB_1_ Cluster Genes

Previous studies have demonstrated that the co-culture of *S. roseolus* and *A. flavus* induces a significant reduction in the AFB_1_ concentration until no detectable level is present, with a slight effect on the growth of *A. flavus* [20]. In the present study, the reduction in the AFB_1_ concentration was the result of the down-regulation of all but one of the genes (*aflT*) in aflatoxin’s gene cluster. The AFB_1_ cluster is internally regulated by the specific transcriptional factors encoded by *aflR* and its coactivator, *aflS.* The interaction between the corresponding proteins is required for the formation of an active complex that triggers AFB_1_ production [21]. As demonstrated, the co-culture condition reduced the concentration of the AflR/AflS complex, leading to the down-regulation of AFB_1_ cluster activation and consequently, to a lower concentration. A transcriptional analysis of the AFB_1_ gene cluster in the co-culture condition demonstrated that genes involved early on in the AFB_1_ enzymatic cascade (i.e., *aflA*, *aflB*, *aflC*) that participate in the construction of the first stable polyketide structure, norsolorinic acid, were less impacted compared to those coding for enzymes involved at the intermediate and latter steps (i.e., *aflK*, *aflM*, *aflO*, *aflP*) [22]. This suggests that the reduced concentration of the AflR/AflS complex might have been consumed at the beginning of the AFB_1_ biosynthesis pathway, interrupting it in the first enzymatic stages and further leading to a significant reduction in the final AFB_1_ concentration. In accordance with this, similar results were obtained using eugenol, a natural extract that reduces the AFB_1_ concentration in *A. flavus* cultures [23].

### 3.2. Effect of S. roseolus on the KA and CPA Concentrations

In this global transcriptomic analysis, the expressions of *kojR* and *dmaT*, which are, respectively, involved in KA and CPA biosynthesis, were also analyzed. The down-regulation of *kojR* was correlated to the decrease of KA concentration in the co-culture condition. These results confirm the report of Marui et al. [24] who demonstrated that the *kojR* gene, encoding a Zn(II)_2_Cys_6_ transcriptional factor, is essential for the activation of the KA cluster and thus, for KA production. In this study, the use of *S. roseolus* simultaneously reduced the AFB_1_ and KA concentrations. On the contrary, other authors observed a reduction in the AFB_1_ concentration with an increase in the KA concentration by using D-glucal, C18:3 fractions, and dioctatin-A [25,26,27]. These results suggest the production of *A. flavus* secondary metabolites depends on the used agent. The expression of the *dmaT* gene in *A. flavus* was also analyzed. This gene, which encodes a dimethylallyltryptophan synthase, is involved in the second step of the CPA biosynthetic pathway and leads to the formation of β-CPA [28]. In this study, the amount of β-CPA was not quantified but the increase of only one intermediate is not enough to lead to an increase in the concentration of the last compound of the biosynthetic pathway (i.e., CPA). Indeed, biosynthetic pathways are complex and from a molecular point of view, all of the involved enzymes operate at the same time in order to take over each intermediate just after their synthesis. An increase in one intermediate does not increase the activity of the involved enzyme during its conversion which might explain why the CPA concentration is the same in both culture conditions. 

### 3.3. Morphological Changes in A. flavus in Co-Culture with S. roseolus

Under co-culture conditions, an increase in the spore amount was observed. This hypersporulated phenotype can be partially correlated to a general over-expression of genes involved in the conidiation process. Among them, the sequential expression of *brlA* and *abaA* plays an important role within the conidiogenesis pathway [29]. *BrlA* encodes an early regulator of fungal development and its activation is necessary and sufficient for conidiophore development [30,31,32]. BrlA also activates AbaA which plays a central role in the differentiation of phialides during the middle stage of conidiophore development after the differentiation of the metulae [33,34]. Therefore, in this study, higher transcription levels of *brlA* were associated with the up-regulation of *abaA* and thus, with the subsequent activation of fungal conidiation. These results are in agreement with those reported by Lv et al., where eugenol, an AFB_1_ inhibitor, induced the increased transcription of *brlA* and *abaA* to activate fungal conidiation in *A. flavus* [35]. Adams et al. also demonstrated that the over-expression of *brlA* in vegetative cells leads to the initiation of cellular transformations resembling those occurring during conidiophore development and conidia production [30]. *BrlA* expression is also linked to the *flb* genes (e.g., *fluG*, *flbA-flbE*) which are individually required for the normal programed switch from hyphae growth to conidiophore development [36]. Specifically, *fluG* is required for an early step in the activation of the sporulation pathway involving the synthesis of an extracellular sporulation inducing factor (ESIF) which is an activator key of conidiation [37]. In co-culture condition, an up-regulation of *fluG* and *flbA* expression levels was observed. These results are in agreement with those reported by Lee and Adams [38] where the over-expression of *fluG* and *flbA* resulted in the over-expression of *brlA*, inducing the formation of conidiophores and allowing sporulation in submerged cultures. The other genes participating in the morphological process analyzed in this study, *veA*, *velB*, *vosA* (belonging to the velvet family), *mtfA*, and *nsdC* (global regulators) were also significantly up-regulated. These genes have also been demonstrated to participate in secondary metabolite production in *Aspergilli* species [39,40,41,42]. Indeed, in this study, differential expressions of these developmental regulators induced significant changes in *A. flavus* morphology that were accompanied by decreased concentrations of AFB_1_ and KA. Another study performed with a natural AFB_1_ inhibitor *Micromeria graeca* in *A. flavus*, also resulted in increased transcription levels of *veA*, *mtfA*, and *nsdC* [12]. Interestingly, morphological aberrances were also observed, for example, phialide formation along the fungal hyphae with an increase of spore quantity. Taken together, these results suggest that the up-regulation of the genes involved in the morphological process induced the hypersporulated phenotype of *A. flavus* in the co-culture with *S. roseolus*. 

### 3.4. Impact of S. roseolus on Fungal Cellular Signalization and the Oxidative Stress Response

In fungi, environmental changes activate signaling pathways as a mechanism of adaptation. Under the co-culture condition, *creA*, a gene encoding a carbon repression regulator, was over-expressed. CreA has also been involved in aflatoxin biosynthesis since several AFB_1_ genes possess CreA sites near to their promoter regions [43]. In fact, a recent study reported that *creA* is highly involved in the mechanism of AFB_1_ inhibition by gallic acid in *A. flavus* [44]. Regarding the other genes involved in cellular signalization, those belonging to the GPCR transmembrane receptors (*gpr* genes), oxylipins dioxygenases (*ppo* genes) and a member of the RAS family (*rasA*) were impacted in *S. roseolus* co-culture. Thus, it has been demonstrated that GPCR members are involved in the oxylipin response and that a proper regulation of the G-protein signalization plays a central role during the activation of signal transduction to surrounding changes as well as regulating secondary metabolite production [45,46,47]. Hence, the presented results suggest that the observed changes in the transcriptional expression levels of the *gpr*, *ppo*, and *ras* genes resulted in a disturbed signal transduction of the G-protein pathway and may negatively impact AFB_1_ biosynthesis. Indeed, among the several GPCRs that were analyzed. *GprA* and *gprP* genes were over-expressed and both were demonstrated as aflatoxin repressors in *A. flavus* [45]. *RasA* expression was also increased and may also be correlated with AFB_1_ reduction since the activation of this GTP-binding protein has been negatively associated with sterigmatocystin production through *aflR* repression in *A. nidulans* [48]. Finally, among the genes involved in the oxidative stress response, *atfB* was the most impacted gene. The down-regulation of this bZIP transcription factor can be associated with both the decreased levels of secondary metabolites and to the stress response. On one hand, due to the fact that AtfB possesses binding sites in the promoters of seven aflatoxin genes [49], its dramatic down-regulation might be hardly implicated in the molecular mechanism leading to AFB_1_ reduction induced by *S. roseolus*. On the other hand, *atfB* is essentially required for *catA* expression [50]; thus, in this study, the decreased levels of *atfB* induced the down-regulation of *catA*, which is involved in conidia stress. Since *catA* disruption in *A. nidulans* produced spores that are sensitive to stress agents such as H_2_O_2_ [51], further studies to evaluate the spore resistance of *A. flavus* under co-culture conditions will be of interest.

## 4. Conclusions

As an alternative to phytopharmaceutical products in the agricultural field, this study provides a proof of concept using biocontrol agents to reduce mycotoxin contamination. *S. roseolus* was used as a biocontrol agent to reduce the aflatoxin B_1_ concentration in *A. flavus* using the co-culture method. Several approaches, such as transcriptomic and morphological studies, were performed to elucidate its molecular mechanism of action. Under the co-culture condition, the main result was the reduction of the aflatoxin B_1_ concentration to undetectable levels. A transcriptomic analysis revealed that the aflatoxin B_1_ biosynthetic pathway was interrupted at an early stage before the norsolorinic acid synthesis—at the first toxic AFB_1_ precursor. The main morphological change in *A. flavus* was the expression of a hypersporulated phenotype accompanied by spore formation directly on fungal hyphae. Taken together, these promising results provide the proof of concept that *S. roseolus* represents an alternative strategy to aflatoxin B_1_, the most highly carcinogenic and mutagenic fungal contaminant.

## 5. Materials and Methods

### 5.1. Chemicals and Reagents

Aflatoxin B_1_ (AF B_1_), cyclopiazonic acid (CPA) and kojic acid (KA) standards were purchased from Sigma-Aldrich (Saint-Quentin-Fallavier, France). Stock solutions were respectively prepared in methanol, ethanol, and water and stored at −18 °C until use. Solvents used for secondary metabolites extraction and High-Performance Liquid Chromatography (HPLC) were of analytical grade quality and were purchased from Thermo-Fisher Scientific (Illkirch, France). The ultrapure water used for HPLC and for the molecular biology procedures was purified at 0.22 µm by an ELGA purification system (ELGA LabWater, High Wycombe, UK).

### 5.2. Strains

The *Streptomyces roseolus* strain used in this study was previously identified [52] and selected for its capacity to reduce the AFB_1_ concentration with a slight reduction in *A. flavus* growth [20] and was maintained on solid ISP2 medium (4 g/L α-d glucose; 10 g/L malt extract; 4 g/L yeast extract; 20 g/L agar). The referenced toxinogenic *Aspergillus flavus* NRRL 62477, producer of AFB_1_, CPA, and KA was isolated from paprika samples harvested from a Moroccan market [53]. Stock cultures of *A. flavus* were maintained on solid Czapek Yeast Extract Agar medium (CYA) (30 g/L sucrose; 5 g/L yeast extract; 15 g/L agar; metal solution).

### 5.3. Culture Conditions

Firstly, the interaction between both strains, *A. flavus* and *S. roseolus*, was performed according to the co-culture method previously described by Sultan and Magan [54] and modified by El Khoury et al. [18]. A cellophane disk (Hutchinson, Chalette-sur-Loing, France) was layered on solid ISP2 medium before the inoculation of strains to allow the separation of mycelium from the culture medium, as previously described by Leite et al. [55]. Then, ten microliters of *A. flavus* calibrated spore suspension (10^6^ spores/mL at 0.05% Tween 80) prepared from a seven-day culture was used to centrally inoculate the solid ISP2 medium. Then, *S. roseolus* mycelium was directly retrieved from a seven-day culture with a sterile steak and was co-inoculated on both sides of the fungal inoculum spot by tracing parallel lines separated from 2 cm from the Petri dish edge. For the control condition, *A. flavus* cultures were inoculated alone. Control and co-culture conditions were conducted in Petri dishes filled with 20 mL of ISP2 medium, and cultures were incubated at 30 °C for four days at a relative humidity of 80% in a Vötsch chamber (Illkirch, France). At the end of the incubation period, on one side, mycelium was separated from the medium by a peel-off cellophane disk and then, quickly frozen and stored at −80 °C until RNA extraction for transcriptomic analysis. One the other side, the medium was collected to perform fungal secondary metabolites extraction, as described below.

### 5.4. Secondary Metabolite Extraction and Quantification by UHPLC/FLD/DAD 

Fungal secondary metabolites were extracted from the entire medium using 25 mL of chloroform and left to macerate for 1 h on a horizontal shaking table set at 200 rpm at room temperature. Extracts were then filtered through a Whatman 1PS phase separator (GE Healthcare Life Sciences, Vélizy-Villacoublay, France) and the organic phase was recovered and evaporated until dryness under a rotavapor set at 60 °C. Samples were resuspended with 500 µL of a water–acetonitrile–methanol mixture (65:17.5:17.5; *v/v/v*) and filtered through 0.45 µm PTFE disk filters (Thermo Scientific Fisher, Villebon-Sur-Yvette, France). The detection of fungal secondary metabolites was performed using a Dionex Ultimate 3000 UHPLC system coupled with a Diode-Array Detector (DAD) and a Fluorescent Detector (FLD) (Thermo Fisher Scientific, Illkirch, France). AFB_1_ was detected using the FLD with 365/440 nm excitation/emission wavelengths, and the UV spectrum was further confirmed by the DAD. CPA and KA were detected using the DAD, which was set at 285 and 265 nm respectively. Analyses of AFB_1_ and KA were performed using a Luna C18 column (3 µm, 200 × 4.6 mm) (Phenomenex, Torrance, CA, USA). A 50 min isocratic mode was delivered with 65% of Eluent A (0.2% acetic acid/water and acetonitrile: 79:21 *v/v*) and 35% of Eluent B (pure methanol). For the CPA analyses, a Luna C18 column was used (3 µm, 150 mm × 2.0 mm, Phenomenex, Torrance, CA, USA). Eluent A consisted of acidified water (0.1% of formic acid) and Eluent B was pure acetonitrile. The following gradient elution program was as follows: 40% B for the first ten minutes, then the percentage of eluent B increased up to 90% within five minutes and remained at this value for ten minutes. Then, the initial conditions were restored within five minutes and remained constant for ten minutes. All the analyses were performed with a flow rate delivered at 0.2 mL/min, a column temperature controlled at 30 °C and an injection volume of 20 µL. The peak identity of each secondary metabolite was confirmed by comparing the UV spectrum and the retention time (min) with the commercial standards (Sigma-Aldrich, Saint-Quentin-Fallavier, France). Quantification of each secondary metabolite was assessed with a standard curve. The limits of quantification (LOQ) were 100 µg/L for AFB_1_, 0.05 mg/L for KA, and 90 mg/L for CPA. The limits of detection (LOD) were 30 µg/L for AFB_1_, 0.01 mg/L for KA, and 20 mg/L for CPA.

### 5.5. Fungal RNA Extraction and Reverse Transcriptase-Polymerase Chain Reaction (RT-PCR)

For the gene expression analysis, RNA was extracted from the collected mycelia which were finely ground under liquid nitrogen. Purification of RNA was performed using the Qiagen RNeasy PlusMinikit (Qiagen, Hilden, Germany) in accordance with the manufacturer’s recommendations. RNA concentrations were quantified using a Nanodrop 2000 spectrophotometer (Thermo Scientific, Illkirch, France) RNA integrity and purity were checked using the Experion RNA analysis kit and software (version 3.20, 2015, BioRad, Marnes-la-Coquette, France). First-strand cDNA synthesis was carried out with an Advantage RT-PCR kit (Clontech, Saint-Quentin-en-Yvelines, France). In accordance with the manufacturer’s instructions, 1 µg of total RNA was synthesized using an oligo dT (5′-GCTGTCAACGATACGCTATAACGGCATGACAGTGTTTTTTTTTTTTTTT-3′).

### 5.6. Quantitative Polymerase Chain Reaction (q-PCR) Analysis

Q-PCR experiments were performed using a ViiA7 Real-Time PCR System (Applied Biosystems, Foster City, CA, USA). The choice of genes analyzed in this study was based on previous works reported by Caceres et al. [23,56] and complementary sequences were added in this study: *kojR* (*F*: ACACCGCGCTGGAGACTATAGA *R*: GTTGTTGAACCTTGTTCGGTCAG); *dmaT* (*F*: GACTGGCCACCTTCTTTGAGC *R*: GCAGTATTTAAGTCCACATCCGGATAG) and *pkaA* (*F*: GCTTCGCAAGTCTCAGCGAT *R*: ACTTCCGCGGCGTAGAACT). For this study, a total of 65 genes were simultaneously analyzed and divided into 4 main categories: (I) 29 genes involved in secondary metabolite biosynthesis, 27 specifically belonging to the AFB_1_ cluster and 2 involved in CPA and KA biosynthesis (*dmaT* and *kojR*); (II) 11 genes related to fungal development, 4 genes linked to the conidiation process (*abaA*, *brlA*, *flbA*, and *fluG*), 4 genes belonging to the velvet family (*veA*, *velB*, *laeA*, and *vosA*), and 3 genes coding for global regulators (*fcr3*, *mtfA*, and *nsdC*); (III) 15 genes involved in external stimuli responses including: 4 genes coding for environmental transcription factors (*areA*, *creA*, *meaB*, and *pacC*) and 11 genes involved in cellular signaling, including G-Protein Coupled Receptors (GPCRs) and sub-units (*gprK*, *gprA*, *gprG*, *gprH*, *gprP*, and *fadA)*, 3 oxylipins (*ppoA*, *ppoB*, and *ppoC*), a member of the RAS family (*rasA*), and a protein kinase A *pkaA;* and (IV) 10 genes involved in the oxidative stress response: *atfA*, *atfB*, *catA*, *cat2*, *sod1*, *mnSOD*, *ap-1*, *msnA*, *srrA*, and *sskA*. For the gene expression experiments, 384-well plates were prepared by an Agilent Bravo Automated Liquid Handling Platform (Agilent Technologies, Santa Clara, CA, USA). The experimental mix consisted of 1 µL of cDNA sample, 2.5 µL of Power SYBR^®^ Green PCR Master Mix (Applied Biosystems, Warrington, UK), and 1.5 µL of the corresponding primer set. Quantitative steps were performed as previously reported by Caceres et al. [23].

### 5.7. Fungal Spore Quantification

The quantity of fungal spores was measured according to the method previously reported by Caceres et al. [55]. Briefly, the entire *A. flavus* mycelium from each Petri dish was submerged in 50 mL of a Tween 80 solution (0.05% *v*/*v* water) to collect fungal spores by scraping. Then, the solution was filtered through sterile gauze and spores were counted using a Thoma cell chamber (Olympus, Rungis, France). Spore quantities from *A. flavus* under control and co-culture conditions were compared.

### 5.8. Microscopic Analysis

Morphological changes of *A. flavus* in co-culture with *S. roseolus* were characterized by Scanning Electronic Microscope (SEM). Samples were directly withdrawn from the co-culture using double-faced metallic adhesive tape. Amelioration of the image signal was achieved by coating samples with gold for 60 s at 10^−1^ mbar using a Scancoat six apparatus (HHV Ltd., Crawley, UK). Electronic images were obtained using a Hitachi TM3000 microscope (Hitachi, Tokyo, Japan), and spectra were recorded using an acceleration voltage of 15 kV.

### 5.9. Statistical Analysis

Student’s *t*-tests followed by a Fischer test on the equality of variance were used to analyze the differences between control and co-culture conditions. The statistical analysis of data was carried out with GraphPad Prism 4 software (GraphPad Software, La Jolla, CA, USA). Differences were considered to be statistically significant when the *p*-value was lower than 0.05. For fungal gene expression, experiments were repeated twice using six biological samples for each condition. Otherwise, three samples per condition were used. Graphical values are represented by mean ± standard error of mean (SEM).
